# Supplementary figures and images for: Improving case-detection of severe wasting among under-five-year-old children in Timor Leste: A secondary analysis of data from the 2020 national cross-sectional food and nutrition survey
Source: PLoS One. 2024 Oct 15;19(10):e0308208. doi: 10.1371/journal.pone.0308208 (PMC11478875; doi:10.1371/journal.pone.0308208)

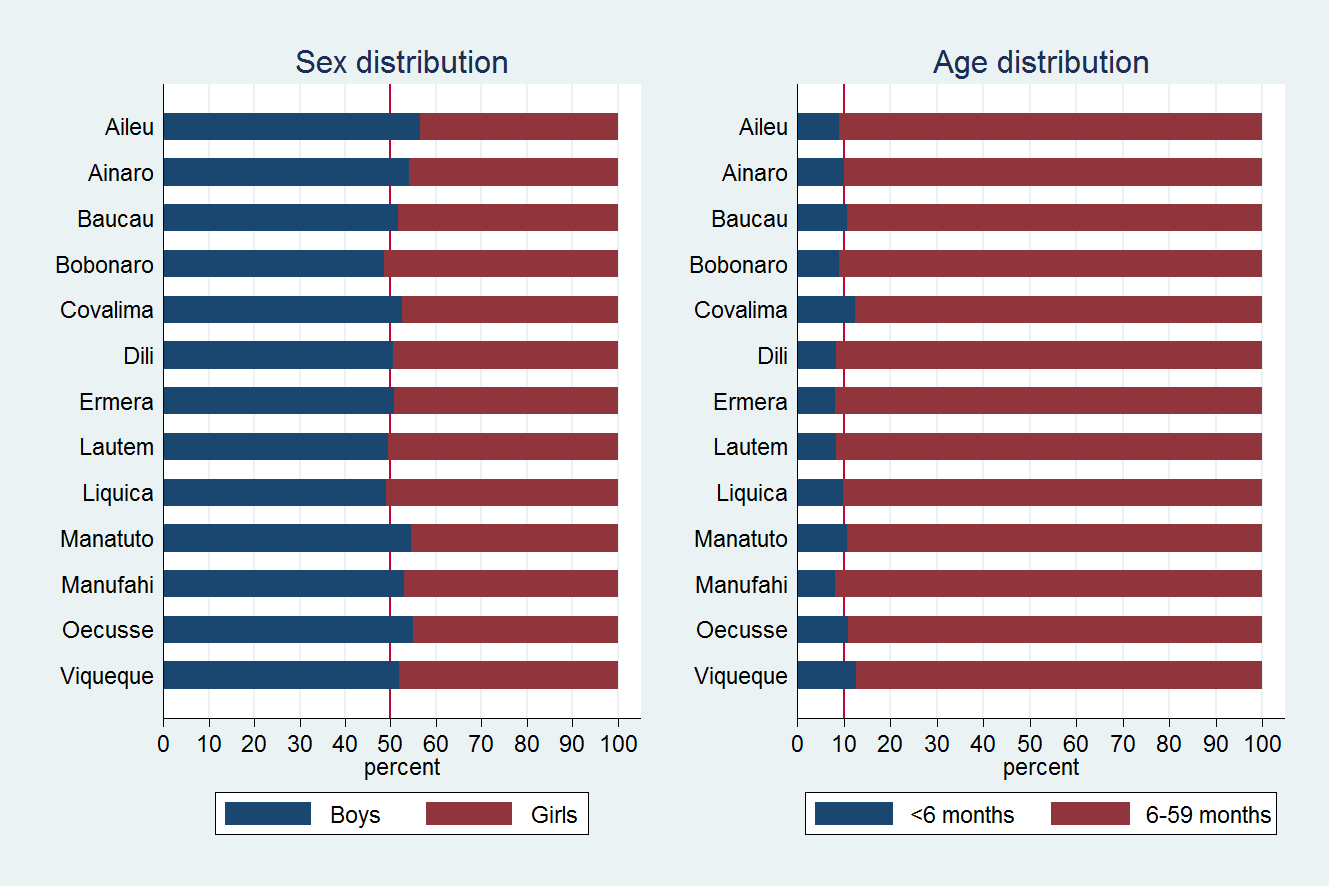

Supplement: S1 Fig — (TIF) [file pone.0308208.s001.tif]
