# Supplementary material for: Improving case-detection of severe wasting among under-five-year-old children in Timor Leste: A secondary analysis of data from the 2020 national cross-sectional food and nutrition survey
Source: PLoS One. 2024 Oct 15;19(10):e0308208. doi: 10.1371/journal.pone.0308208 (PMC11478875; doi:10.1371/journal.pone.0308208)
Supplement: Attachment — Submitted filename: Response to reviewers1.docx [file pone.0308208.s003.docx]

PONE-D-24-05436

Improving case-detection of severe wasting among under-five-year-old children in Timor-Leste: A secondary analysis of data from the 2020 national cross-sectional food and nutrition survey

PLOS ONE

Dear editors and reviewers,

Thanks for your investment in reviewing our manuscript and for the constructive comments and suggestions. We have considered all of them and made the required adjustments. .

As requested by the reviewers, we have restructured the discussion section to include requested additional information. This has resulted in a much longer discussion section, given that we had to add new paragraphs. Also, we have removed some references and added new to address the concerns raised related to our references list. We made several changes on the reference list including corrections of incomplete references, removal of some references, and addition of new references. The number of references has increased from 93 to 104.

Please, find below the more detailed point-by-point responses to your comments and queries.

**Journal Requirements:**

When submitting your revision, we need you to address these additional requirements.

1. Please ensure that your manuscript meets PLOS ONE's style requirements, including those for file naming.

Thank you for the recommendation. This has been done.

2. Please note that your Data Availability Statement is currently missing [the repository name and/or the DOI/accession number of each dataset OR a direct link to access each database]. If your manuscript is accepted for publication, you will be asked to provide these details on a very short timeline. We therefore suggest that you provide this information now, though we will not hold up the peer review process if you are unable.

Thank you very much. We have now provided the raw data as supporting information

3. PLOS requires an ORCID iD for the corresponding author in Editorial Manager on papers submitted after December 6th, 2016. Please ensure that you have an ORCID iD and that it is validated in Editorial Manager. To do this, go to ‘Update my Information’ (in the upper left-hand corner of the main menu), and click on the Fetch/Validate link next to the ORCID field. This will take you to the ORCID site and allow you to create a new iD or authenticate a pre-existing iD in Editorial Manager. Please see the following video for instructions on linking an ORCID iD to your Editorial Manager account: <https://www.youtube.com/watch?v=_xcclfuvtxQ>.

We have provided the ORCID. Also, we have linked the paper to our ORCID

4. We note that the grant information you provided in the ‘Funding Information’ and ‘Financial Disclosure’ sections do not match. When you resubmit, please ensure that you provide the correct grant numbers for the awards you received for your study in the ‘Funding Information’ section.

Corrections have been made.

5. Please include captions for your Supporting Information files at the end of your manuscript, and update any in-text citations to match accordingly. Please see our Supporting Information guidelines for more information: <http://journals.plos.org/plosone/s/supporting-information>.

We apologize for the omission. We have added the section and implemented your recommendation.

6. Please review your reference list to ensure that it is complete and correct. If you have cited papers that have been retracted, please include the rationale for doing so in the manuscript text, or remove these references and replace them with relevant current references. Any changes to the reference list should be mentioned in the rebuttal letter that accompanies your revised manuscript. If you need to cite a retracted article, indicate the article’s retracted status in the References list and also include a citation and full reference for the retraction notice.

This has been done.

7. While revising your submission, please upload your figure files to the Preflight Analysis and Conversion Engine (PACE) digital diagnostic tool, <https://pacev2.apexcovantage.com/>. PACE helps ensure that figures meet PLOS requirements. To use PACE, you must first register as a user. Registration is free. Then, login and navigate to the UPLOAD tab, where you will find detailed instructions on how to use the tool. If you encounter any issues or have any questions when using PACE, please email PLOS at [figures@plos.org](mailto:figures@plos.org). Please note that Supporting Information files do not need this step

This has been done. We have ensured that all the references are written as per EndNotes formatting.

**Reviewers' comments:**

Reviewer #1:

Dear author,
This work is very fine

Thank you very much for the compliment

Please discuss in detail with similar study conducted so far to show the trend.

Thank you for this comment. We have revised the discussion section by revising several paragraphs and adding new ones to implement this recommendation (pages 22 to 26, lines 352 to 456).

Please update old references.

In the academic and scientific community, there isn't a strict consensus on the age of articles to be cited in a scientific paper. In our paper, we followed the general rule of thumb of prioritizing the relevance and avoiding citation of secondary sources (https://apastyle.apa.org/blog/outdated-sources-myth). We only cited old articles that we consider seminal or foundational to the topic being discussed. Because of this comment, we reviewed journal guidelines to check for any specific requirements regarding the age of the references and noted that the journal has no specific recommendations. Also, we would like to emphasize the fact the paper is not covering a faster-paced field and that there is no a specific rule regarding the age of the article to be cited for the covered topic. Nevertheless, as a result of the revision of the discussion section, we have included several new recent references.

Please check once the grammatical and any technical error in the manuscript.

Thank you for your careful review of the paper and for spotting the existence of typos. We have reviewed the entire manuscripts and we have corrected all grammar and spelling errors.

Reviewer #2:

Introduction
Lines 56, 57, and 66 need references.

Thanks for this comment. We have added the requested references.

In line 72 write the word countries instead of counties.

Thanks for spotting the spelling error. We have corrected the error.

In line 77, what does IMAM mean? Abbreviate it. The author abbreviates it at line 120. Kindly abbreviate it at the start.

Thank you for spotting this omission. Correction has been made both in lines 77-78 and lines 121-122.

Sometimes authors capitalize the word ‘Malnutrition’, and sometimes write it in small letters like ‘malnutrition’. Check lines 83 and 85. Use one format.

Thanks for spotting this. We have made the correction (page 4, line 84).

The authors used around 47 references older than 5 years. As it's well-known that a research paper should use the last 5 years of literature, to obtain the most relevant information. The percentages of the last 5 years of references were 49.5 % (46 references) while old references were 50.5% (47 references). Kindly use updated references.

Thanks for this comment. However as indicated above when responding to similar comment from reviewer 1 we think that this metric is not applicable for the research field of this paper. For instance, even WHO commissioned literature review (well-funded and conducted by in parallel by several teams of world class experts in the field) never fulfilled this criterion because this is not faster-paced field (https://files.magicapp.org/guideline/a3fe934f-6516-460d-902f-e1c7bbcec034/published_guideline_7330-1_1.pdf). We would like to stress that the metric you are referring to is based on journals selectively publishing papers of faster-paced research fields (<https://nxref.com/recent-research-works-for-good-referencing/>) and that this metric cannot be applied to all scientific fields. When the use of such metric will be mandatory will be needed for all the scientific topics, a specific metric will have to be developed for each field.

Discussions
In line 353 the sentence does not end with full stop. Check punctuation marks in the whole manuscript.

Thanks for spotting the error. We have rechecked the entire manuscript for punctuation.

In line 362, there are 2 full stops at the end of the sentence. Please correct it.

Thank you very much for spotting it. We have rechecked the entire manuscript for punctuation.

Also, check grammar mistakes.

Thanks. We have implemented this recommendation.

Conclusion
Please add further directions (e.g., further studies on what should be considered).

Thank you for highlighting the need to propose further research. We have already recommended that studies to confirm the suitability of the proposed case-definitions and a study to document the short and long-terms outcomes of severely wasted children not identified by these case-definitions, be conducted (page 28, lines 515-517). In response to this suggestion, we have added additional propositionsof further research (Page 28-29; lines 517-521)

Add one simple paragraph for easy understanding of the common person.

We have reviewed the journal formatting guidelines and we can confirm that such paragraph is not required per the journal style. Thus, this recommendation is not implemented.

Please check the whole manuscript carefully before resubmitting.

Thank you, we have implemented this recommendation.

.

.
